# Zootherapeutic uses of wildmeat and associated products in the semiarid region of Brazil: general aspects and challenges for conservation

**DOI:** 10.1186/s13002-018-0259-y

**Published:** 2018-09-17

**Authors:** Wedson Medeiros Silva Souto, Raynner Rilke Duarte Barboza, Hugo Fernandes-Ferreira, Arnaldo José Correia Magalhães Júnior, Julio Marcelino Monteiro, Érika de Araújo Abi-chacra, Rômulo Romeu Nóbrega Alves

**Affiliations:** 1 Department of Biology, Laboratory of Zoology, Wildlife Use and Conservation (ZUCON), Federal University of Piaui (UFPI), Campus Ministro Petrônio Portella, Teresina, Piaui Zip code/CEP 64049-550 Brazil; 2 Programa de Pós-Graduação em Ciências Biológicas (Zoologia), Departamento de Sistemática e Ecologia, Federal University of Paraiba (UFPB), João Pessoa, Paraiba CEP 58059-970 Brazil; 3 Department of Biology, State University of Paraiba (UEPB), Av. Baraúnas n. 351, Campina Grande, Paraiba CEP 58109-753 Brazil; 4 State University of Ceara (UECE), Faculdade de Educação, Ciências e Letras do Sertão Central, Quixadá, Ceará CEP 63900-000 Brazil; 5 Natural Sciences Course, Federal University of San Francisco Valley (UNIVASF), São Raimundo Nonato, Piaui CEP 64770-000 Brazil; 6 Biological Sciences Course, UFPI, Campus Amílcar Ferreira Sobral (CAFS), Floriano, Piaui CEP 64800-000 Brazil; 7 Department of Parasitology and Microbiology (DPM), Federal University of Piaui (UFPI), Campus Ministro Petrônio Portella, Teresina, Piaui Zip code/CEP 64049-550 Brazil

**Keywords:** Neotropical fauna, Wildlife conservation, Traditional medicine, Wildlife trade, Bushmeat

## Abstract

**Background:**

Hunting wildlife for medicinal purposes is a widespread practice throughout Brazil; however, studies about the animals used for zootherapeutic practices have been performed almost exclusively with traders (herbalists) and end consumers, and not hunters. This makes it difficult to completely understand the market chain, trade strategies, and drivers of this practice. The present study investigated the species hunted or trapped for traditional medicinal uses by collecting data about the use and trade of the zootheurapeutic species.

**Methods:**

We collected data through semi-structured questionnaires complemented by free interviews and informal conversations with hunters in five municipalities of semiarid region of the NE Brazil. We calculated the Use-Value (UV) index to determine the relative importance of each species reported by interviewees. The Multiple Linear Regression model was used to assess the influence of socioeconomic factors (age, schooling, residence zone, trade of zootherapeutic species) on species richness exploited by hunters.

**Results:**

Hunters reported a significant richness of species (*n* = 39) intentionally or opportunistically captured for use as remedies for treatment of 92 diseases or conditions in humans or livestock. Respondents also reported trade strategies that were well-organized and quickly directed the selling of wild animals or byproducts via modern technology. We found a weak positive relationship only between species richness and hunters’ age via MLR model.

**Conclusions:**

The hunting and use of wild species for medicinal purposes are culturally disseminated activities among hunters. Our results demonstrate the importance of studying hunters in order to understanding the dynamics of bushmeat exploitation and to develop more efficient strategies for wildlife use and conservation.

## Background

Throughout the tropics, humans exploit wild animals for many purposes, including for medicinal products [1–3]. The reasons for using such zootherapeutic products by urban or peri-urban populations are diverse [4–7], and while for many people it is due to the lack of easy access to allopathic remedies, it is also true that the use of wild animal parts can be strongly motivated by beliefs and traditions [8–10].

The expansion of global markets and access to modern medicine seem to have not led to a decrease in the demand for medicinal wildlife products [9, 11]. In fact, several studies have shown that urbanization and the growth of trade have actually expanded the exploitation and trafficking of wildlife [12, 13]. Additionally, the widespread exploitation of wildlife by practitioners of traditional medicine has direct implications for culture, bioprospecting, public health, and biodiversity, given the large number of species used and the growing number of taxa threatened by this type of trade [14].

Nearly 350 animal species (176 terrestrial vertebrates) are involved in the traditional Brazilian pharmacopeia [15]. However, this number is probably an underestimate in light of new research regarding zootherapy in several parts of Brazil, and the great cultural and faunal diversity of this continent-sized country [7, 16, 17]. The Northeast Region of Brazil, in particular, assuredly possesses a significant diversity of species that are hunted as sources for popular remedies and which have yet to be reported in the literature. This is because, first of all, hunting of wildlife species for medicinal purposes is a very common practice in the semiarid area of the Northeast Region of Brazil, and zootherapeutic remedies play significant roles there in the treatment of human and livestock diseases [18, 19]. Second, the semiarid area of the Northeast Region of Brazil (Caatinga phytogeographical domain) possesses a rich diversity of terrestrial vertebrates, including 511 species of birds, 156 mammals and 175 reptiles and amphibians, in parallel with cultural richness among the local human inhabitants and their diverse interactions with wildlife [20]. This scenario suggests that the region is extremely suitable for research on wildlife hunting for medicinal, as well as other, purposes.

Available knowledge concerning the use and trade of wildlife in traditional medicine has largely come from herbalists, livestock keepers, market vendors, or end consumers in urban and rural areas (e.g., [4, 19]), and not from hunters themselves. Furthermore, most of these studies took place in isolated communities (often without access to complete health services) or in public markets where medicinal products derived from animals are sold [19, 21]. Therefore, we chose to investigate the acquisition of medicinal animals by urban and peri-urban hunters in the semiarid area of the state of Paraiba in the Northeast Region of Brazil. We assumed that hunters, being users of animal-based medicines and the individuals directly involved in capturing wild specimens, would possess detailed information concerning the species considered to be sources of zootherapeutic products and the strategies for their storage and distribution.

Therefore, we sought to identify the species of wild terrestrial vertebrates used by hunters for medicinal purposes in the semiarid area of the Northeast Region of Brazil, in order to record which diseases are treated with animal-based remedies, verify the motivations for zootherapeutic animal hunting and to identify the local distribution of animal-based remedies within the market chain. Our main hypothesis is that the richness of species richness exploited by hunters for medicinal purposes is influenced by socioeconomic factors. Secondly, we hypothesize that the local use and trade of animals for medicinal purposes has, despite enforcement strategies, adapted over time into illegal exploitation supported by social networks and the expansion of technological resources.

## Methods

### Study areas

**Fig. 1:**
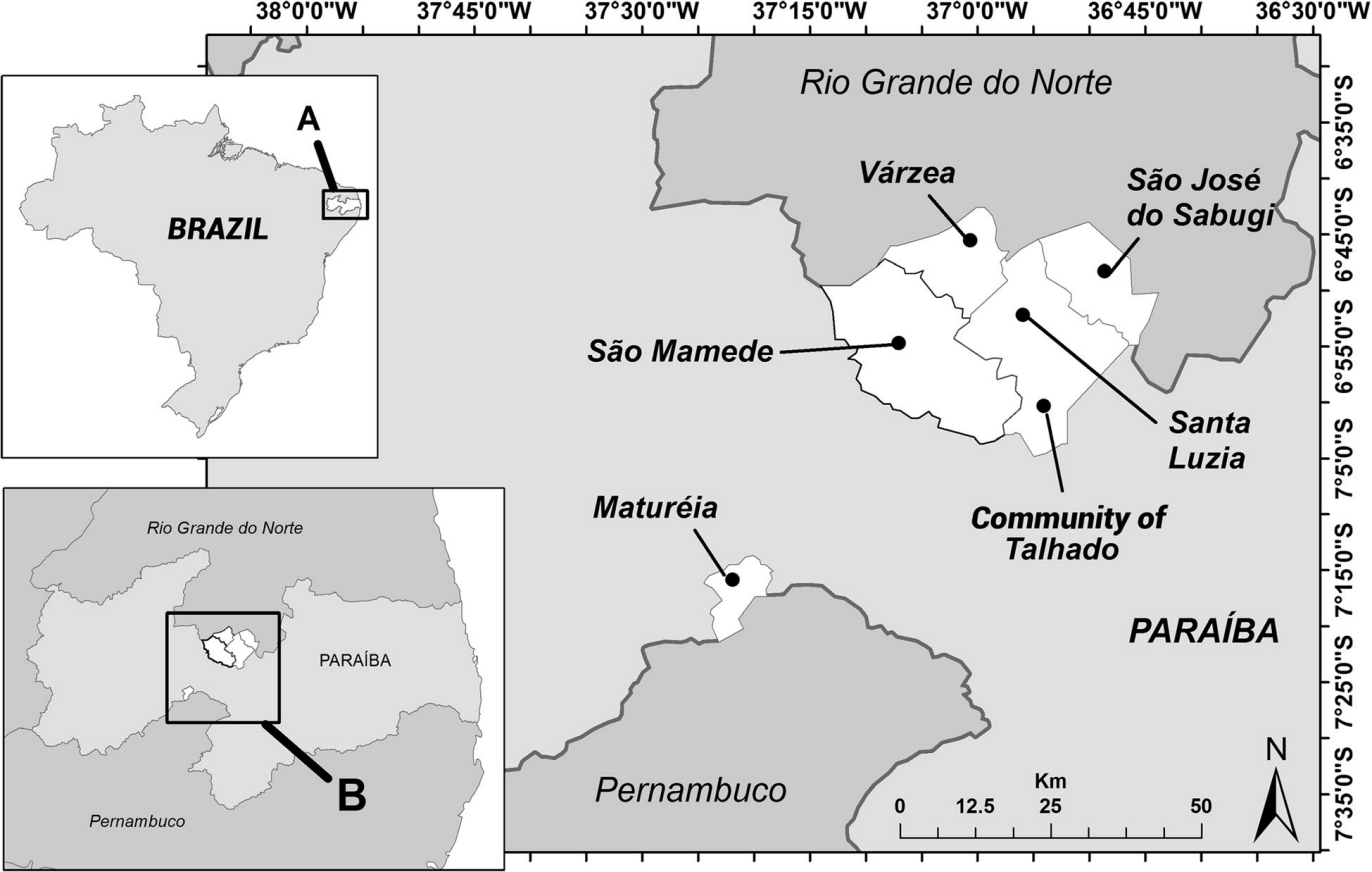
Map of study areas

The present study took place in five municipalities (Maturéia, Santa Luzia, São José do Sabugi, São Mamede, and Várzea) in the semiarid area of the state of Paraiba, Brazil. Data were also collected in Community of Talhado, a traditional Afro-descendant peri-urban community located in the municipality of Santa Luzia, approximately 25 km traveling distance from the nearest urban area (Fig. 1).

According to data from IBGE (*Instituto Brasileiro de Geografia e Estatística*) for 2010, Santa Luzia had 14,719 inhabitants, followed by São Mamede (7748 inhabitants), São José do Sabugi (4010), and Várzea (2504) [22]. Community of Talhado had approximately 120 residents based on Family Health Program (PSF) data (personal obs.). The human development index was medium in all municipalities in 2010, ranging from 0.646 in São Mamede to 0.676 in Santa Luzia [22, 23]. Except for São José do Sabugi, all municipalities had a basic hospital and broad access to allopathic medicine through pharmacies and community health centers (CHC). Despite having a CHC, local residents of the Community of Talhado rely on medical services and resources of the Santa Luzia urban area.

The climate of the municipality of Maturéia is warm and semi-humid (AW’) with summer rains between January and May [24]. The region is characterized by the presence of rocky outcrops (granite and gneiss) and sub-xerophytic semi-deciduous vegetation known as “montane forest” [25]. The local vegetation has floristic elements characteristic of humid forest and the Caatinga [25]. The climate of the other studied municipalities is semiarid tropical (BSh) with a rainfall index of 800 mm/year [26] and native vegetation consisting of hyper-xerophytic caatinga [27, 28]. The local caatinga fauna comprises at least 510 species of birds and 150 species of mammals [20].

### Data collection

Fieldwork took place during three time periods of 2010 and 2011. Information concerning hunting and the use of vertebrates for medicinal purposes was gathered using semi-structured questionnaires complemented by free interviews and informal conversations. Questionnaires covered several key points: (1) whether the interviewees (hunters) captured animals for medicinal purposes; (2) the local names of the zootherapeutic animals; (3) hunting/trapping methods used for taking target species; (4) diseases/conditions treated with zootherapeutics; (5) the manners of preparation and administration of animal-based medicines; (6) possible restrictions and/or adverse effects; (7) spiritual aspects linked to the use of medicinal animals; and (8) aspects of (illegal) commercialization of medicinal animals and their byproducts.

We employed opportunistic sampling [29, 30] to contact respondents because hunting activities and wildlife uses are illegal in Brazil (Brazilian law 9.605/98) and most people tend to be reluctant (or to even refuse) to participate in this type of research [31]. The total sample consisted of 257 respondents in six places of Paraíba State, Northeast Brazil: 58 hunters from São Mamede, 48 from São José do Sabugi, 43 from Várzea, 41 from Community of Talhado, 34 from Maturéia, and 33 from Santa Luzia.

Prior to the execution this study, the Research Ethics Committee of Lauro Wanderley University Hospital (CEP/HULW – Federal University of Paraiba) approved two projects that provided support for this research (Registration numbers CEP/HULW 103/10 and 104/10, CAAE numbers 0146.0.126.000-10 and 0177.0.126.000-10).

### Species identification, conservation status, and average weight

Species were identified based on (1) analyses of individuals (or body parts) donated by interviewees; (2) analyses of photographs of animals taken during the interviews or while accompanying hunting activities or trade operations; and (3) tracing vernacular names with the help of taxonomists familiar with the local wildlife. The identification of birds was also facilitated by the use of specialized literature [32] and reliable digital sources [33].

The classification and nomenclature of taxa follow the Brazilian Committee of Ornithological Registration for birds [34], Brazilian Society of Herpetology for reptiles [35] and amphibians [36], and the Catalogue of Life version 2016 [37] for mammals. The conservation status of the recorded species follows the IUCN Red List 2017-3 [38].

In order to determine which taxa, in general terms, provide the most potential biomass for use as source of medicinal products, we defined the average body weight of the exploited species using the following reliable sources for amphibians [39], reptiles [39–43], birds [32, 33], and mammals [44, 45]. As several times we only had access to by-parts of wild animals, literature data were the only way to obtain the weight of target species.

### Data analysis

We employed the Use-Value (UV), an ethnobiological index adapted from Phillips and Gentry [46] by Rossato et al. [47], to elucidate the relative importance of each species reported. Use-Value is calculated by UV = ∑*U*/*n*, where *U* is the number of citations per species and *n* is the number of informants/interviewees.

We assessed the influence of socioeconomic factors (age, schooling, residence zone, trade of zootherapeutic species) on species richness exploited by hunters using a multivariate analysis (Multiple Linear Regression model, MLR). The categorical variables of schooling, residential zone of hunter, and trade of zootherapeutic species by hunter were converted into dummy variables (0 or 1) for the model. The statistical analyses were performed using BioEstat version 5.3 [48]. The level of significance adopted was 5% (*p* < 0.05).

## Results

### Socioeconomics

The age of the hunters interviewed ranged from 14 to 86, with a mean of 42.82 ± 17.68 (SD). Most respondents (*n* = 120, 46.7%) were adults between 30 and 60 (Table 1), although there were a significant number of active hunters over 60.

**Table 1:** Hunters’ socioeconomic aspects

Key socioeconomic aspects	Locations						Total
	MAT	SJS	SL	SM	CT	VA	
Age							
Less than 30 years old (yo)	6	23	11	20	9	8	77 (29.96%)
30–39 yo	3	7	11	12	7	7	47 (18.29%)
40–49 yo	7	8	5	7	7	8	42 (16.34%)
50–59 yo	4	5	4	8	8	6	35 (13.62%)
60 or older	14	5	2	11	10	14	56 (21.74%)
Education level							
Low							
Illiterate/semi-literate	14	7	4	20	19	17	81
Total “low level”							81 (31.52%)
Medium							
Elementary school/Junior High School (from 1st to 7th grades) incomplete	19	28	23	25	15	14	124
Junior High School complete (8th grade finished)	1	2		3	4	2	12
Total “medium level”							136 (52.92%)
High							
Secondary school incomplete		6	2	6	1	3	18
Secondary school complete		5	4	4	2	6	21
Superior education incomplete or complete						1	1
Total “high level”							40 (15.56%)
Personal income							
Up to a minimum wage (≤ 320.6 USD)	6	3	4	8	7	2	30 (11.67%)
1–3 minimum wage (> 320.6 USD, < 961.8 USD)	26	41	23	48	33	29	200 (77.82%)
>3 minimum wage (> 961.8 USD)	2	4	6	2	1	12	27 (10.51%)
Sell species for traditional medicine							
Yes	27	32	30	47	22	31	189 (73.54%)
No	7	16	3	11	19	12	68 (26.46%)
Receive “*Bolsa família*”							
Yes	20	31	24	38	31	20	164 (63.81%)
No	14	17	9	20	10	23	93 (36.19%)
Residence zone							
Urban	27	41	28	33		23	152 (59.14%)
Peri-urban	7	7	5	25	41	20	105 (40.86%)
House							
Own	33	34	23	46	41	33	210 (81.71%)
Rented home		6	3	1		3	13 (5.06%)
Another situation	1	8	7	11		7	34 (13.23%)
Motor vehicle at home							
Yes	19	29	24	34	19	29	154 (59.92%)
No	15	19	9	24	22	14	103 (40.08%)

Study sites: *MAT* Maturéia, *SJS* São José do Sabugi, *SL* Santa Luzia, *SM* São Mamede, *CT* Community do Talhado, *VA* Várzea

The personal income of the interviewees was low or average for the region. A total of 200 respondents had personal monthly income greater than one, and less than three, times the minimum wage (> 320 and < 960 USD). Most of the families of the hunters (63.8%) received conditional cash transfers from the federal government (*Bolsa família*) (Table 1). According to the United Nations Development Programme [23], the Brazilian Human Development Index (HDI) improved from medium (0.683) in 2000 to high (0.742) in 2011, which was indirectly perceptible by the fact that 59.9% (*n* = 154) of the interviewees had motorized vehicles at their residences (Table 1), and almost all of them had mobile/smart phones (92.6%; *n* = 238).

### Medicinal bushmeat species and zootherapeutic uses

A total of 39 terrestrial vertebrates distributed among 27 families were reported by the hunters as sources of zootherapeutic remedies (Table 2). Mammals (with 16 spp.) were the most represented group, followed by birds (13 spp.), reptiles (8 spp.), and amphibians (2 spp.), with no more than six species (*n* = 15.4%) reported as being used at any one location. We found eight new records of species hunted or trapped for traditional medicine: *Bothrops erythromelas* Amaral, 1923, *Sarkidiornis sylvicola* Ihering & Ihering, 1907, *Columbina minuta* (Linnaeus, 1766), *Columbina picui* (Temminck, 1813), *Columbina squammata* (Lesson, 1831), *Columbina talpacoti* (Temminck, 1810), *Chlorostilbon lucidus* (Shaw, 1812), *Sapajus libidinosus* (Spix, 1823), and *Thrichomys laurentius* Thomas, 1904.

**Table 2:** Wild animal species hunted for traditional medicine in semiarid of NE Brazil

Class/Family/*Species*/“Local name”, popular name (En-US)	Average weight (kg)	Parts used for medicinal purposes	Disease (or illness) treated	IUCN Red List	UV
Amphibians (Amphibia)					
Bufonidae					
*Rhinella jimi* (Stevaux, 2002)—“Cururu”	0.2	Leather, fat (*banha*), viscera	Itches, “esponja de cavalo” (Dermal wounds brought about by infestation of larvae of *Habronema muscae*), inflammations, “estrepes” (suck a splinter out of skin), wounds, cracked feet, hangnail	LC	0.05
Leptodactylidae					
*Leptodactylus vastus* A. Lutz, 1930—“jia”, Northeastern Pepper Frog	~ 1	Meat	Eczema, sore throat, swellings	LC	0.01
Reptiles (Reptilia)					
Boidae					
*Boa constrictor* Linnaeus, 1758—“cobra de veado”, “jibóia”, Boa	5.6	Fat (*banha*)	Arthritis, pains, to promote hair growth in areas affected by burns, fractures, wounds, Herpes zoster (“cobreiro”), infections, sore throat, laryngitis, muscle injuries, dermal nodules, omphaloarteritis (“caruara de bezerro”), spinal disorders, “estrepes” (suck a splinter out of skin), rheumatism, cracked feet		0.47
Chelidae					
*Mesoclemmys tuberculata* (Lüderwaldt, 1926)—“Cágado do nordeste”, “cágado d’água amarelo”, Tuberculate Toad-headed Turtle	n.o.	Meat, fat (*banha*), eggs	Diphtheria, headache, toothache, earache, chest pain, wounds, furuncle, gastritis, sore throat, hemorrhoids, swellings, spinal disorders, eye problems (especially blindness), “estrepes” (suck a splinter out of skin), rheumatism, deafness		0.53
*Phrynops tuberosus* (Peters, 1870)—“cágado d’água”, Geoffroy’s Side-necked Turtle	1.03	Meat, fat (*banha*), eggs	Diphtheria, headache, toothache, earache, chest pain, wounds, furuncle, gastritis, sore throat, hemorrhoids, swellings, spinal disorders, eye problems (especially blindness),"estrepes” (suck a splinter out of skin), rheumatism, deafness		0.52
Iguanidae					
*Iguana iguana* (Linnaeus, 1758)—“Camaleão”, Common Green Iguana	2.6	Whole specimen, meat, leather, fat, bone	Lack of appetite, pains in general, appendicitis, kidney stone, prostate cancer, to promote hair growth in areas affected by burns, diabetes, toothache, bone pain, eczema, wounds, mouth sores, gastritis, flu, “impinge” (ringworm), inflammations, leprosy in dogs, dermal nodules, snake bites, throat problems, rheumatism, “estrepes” (suck a splinter out of skin), hoarseness, deafness, tuberculosis		0.44
Teiidae					
*Salvator merianae* (Duméril & Bibron, 1839)—“lagarto Teju”, Tegu Lizard	4	Leather, liver, fat (*banha*)	Lack of appetite in dogs and pigs, dores de ouvido, toothache, diphtheria (“crupe”), fever, wounds, mouth sores, gastritis, flu, swellings, inflammations, sore throat, otitis, swellings, snake bites in humans and dogs, throat problems, hoarseness, “estrepes” (suck a splinter out of skin), rheumatism, sinusitis, deafness, tumors	LC	1.09
Tropiduridae					
*Tropidurus hispidus* (Spix, 1825)—“lagartixa de lajedo”	0.025	Whole specimen, leather, liver, bone, viscera	Alcoholism, wounds, hernia, micoses, throat problems, “pano branco” (pityriasis versicolor), “tosse braba”, verrugas		0.04
Viperidae					
*Bothrops erythromelas* Amaral, 1923—“Jararaca malha de cascavel”, “jararaca verdadeira”,	0.2	Whole specimen	Cancer	LC	0.01
*Crotalus durissus* Linnaeus, 1758—“cascavel”, South American Rattlesnake	2.4	Fat (*banha*), rattle (*maracá*)	Pains, arthritis, asthma, cancer, eczema, erysipelas, wounds, swellings, sore throat, uterine inflammation, “mau-olhado” (evil eye), “estrepes” (suck a splinter out of skin), dermal nodules, omphaloarteritis (“caruara de bezerro”), osteoporosis, snake bites, throat problems, spinal disorders, rheumatism	LC	0.52
Aves					
Anatidae					
*Sarkidiornis sylvicola* Ihering & Ihering, 1907—“putrião”, Comb Duck	2	Caruncúla (a large fleshy comb protruding from their upper mandible)	Wounds	LC	0.01
Columbidae					
*Columbina minuta* (Linnaeus, 1766)—“rolinha-cambuta”, “rolinha cabocla”, Plain-breasted Ground Dove	0.034	Meat	Lack of appetite, sickness of pregnant women	LC	0.02
*Columbina picui* (Temminck, 1813)—“rolinha-branca”, Picui Ground Dov	0.052	Meat, feces	Lack of appetite, sickness of pregnant women, deafness	LC	0.03
*Columbina squammata* (Lesson, 1831)—“rolinha-cascavelhinha”, Scaled Dove	0.054	Meat	Sickness of pregnant women	LC	0.004
*Columbina talpacoti* (Temminck, 1810)—“rolinha-caldo-de-feijão”, Ruddy Ground Dove	0.047	Meat	Lack of appetite, sickness of pregnant women	LC	0.01
*Leptotila rufaxilla* (Richard & Bernard, 1792)—“juriti”, Gray-fronted Dove	0.149	Meat, gizzard membrane	Lack of appetite, sickness of pregnant women, snake bites in dogs, “terçol” (inflammation of the Zeis and Mol glands)	LC	0.02
Corvidae					
*Cyanocorax cyanopogon* (Wied, 1821)—“cancão”, White-naped Jay	0.175	Whole specimen	Asthma, “mau-olhado” (evil eye)	LC	0.05
Cracidae					
*Penelope jacucaca* Spix, 1825—“Jacu”, White-browed Guan	~ 1	Feathers	Epilepsy	VU	0.02
Cuculidae					
*Crotophaga ani* Linnaeus, 1758—“anum-preto”, Smooth-billed Ani	0.149	Meat	Asthma	LC	0.02
Podicipedidae					
*Tachybaptus dominicus* (Linnaeus, 1766)—“mergulhão-pequeno”, “mergulhão”, “mergulhão-preto”, Least Grebe	0.155	Gizzard membrane	Improve eyesight	LC	0.003
Tinamidae					
*Nothura boraquira* (Spix, 1825)—“codorniz”, “codorniz do papo-branco”, White-bellied Nothura	0.250	Feathers	Asthma, blindness, sickness of pregnant women, convulsion, earache, “scare bats”, breathlessness, weakness in women at postpartum, snake bites	LC	0.35
*Nothura maculosa* (Temminck, 1815)—“lambú espanta-boiada”, “lambú-de-capoeira”, Spotted Nothura	0.300	Feathers	Snake bites	LC	0.04
Trochilidae					
*Chlorostilbon lucidus* (Shaw, 1812)—“beija-flor-verde”, Glittering-bellied Emerald	0.003	Nest	Earache	LC	0.02
Mammals (Mammalia)					
Canidae					
*Cerdocyon thous* (Linnaeus, 1766)—“raposa”, Crab-eating Fox	7.4	Meat, tail, leather, fat (banha), bones	Aftosa, asthma, erysipelas, “mau-olhado” (evil eye), wounds, uterine inflammations, hemorrhoids, inflammations in general, sore throat, swellings, “to protect of bat attacks”, “estrepes” (suck a splinter out of skin), cracked feet, rheumatism	LC	0.48
Caviidae					
*Galea spixii* (Wagler, 1831)—“preá”, Spix’s Yellow-toothed Cavy	0.350	Meat, teeth, bones	To facilitate tooth eruption in children, cracked feet, ear problems	LC	0.14
*Kerodon rupestris* (Wied-Neuwied, 1820)—“mocó”	0.750	Meat, “coalho” (part of the stomach), fat, feces, fel, bones, gall bladder	Lack of appetite, alcoholism, anemia, asthma, kidney stone, prostate cancer, malnutrition, earache, weakness, gastritis, urethra infections, hernia, osteoporosis, sickness of pregnant women, kidney problems, indigestion, rheumatism, measles, facilitate tooth eruption in children	LC	0.31
Cebidae					
*Sapajus libidinosus* (Spix, 1823)—“macaco prego”, Bearded Capuchin	3.1	Meat	Osteoporosis	LC	0.01
Cuniculidae					
*Cuniculus paca* (Linnaeus, 1766)—“paca”, Spotted Paca	9.35	Gall bladder	Rheumatism	LC	0.01
Dasypodidae					
*Dasypus novemcinctus* Linnaeus, 1758—“tatu-verdadeiro”, Nine-banded Armadillo	4.5	Tail, liver, dermal plates	Asthma, earache, improve the olfaction of hunting dogs, snake bites, “mau-olhado” (evil eye), deafness	LC	0.17
*Euphractus sexcinctus* (Linnaeus, 1758)—“tatu-peba”, Six-banded Armadillo	4.85	Tail, meat	Pains, earache, furuncles, “estrepes” (suck a splinter out of skin), wounds, deafness, “mau-olhado” (evil eye)	LC	0.03
Didelphidae					
*Didelphis albiventris* Lund, 1840—“timbú”, White-eared Opossum	1.62	Meat, fat	Wounds, weakness	LC	0.02
Echimyidae					
*Thrichomys laurentius* Thomas, 1904—“punaré”	0.282	Feces	Diarrhea	DD	0.004
Felidae					
*Leopardus pardalis* (Linnaeus, 1758)—“gato-maracajá”, Ocelot	~ 8	Tail, fat	Headache, throat problems, spinal disorders, wounds, “to protect of bat attacks”	LC	0.05
*Leopardus tigrinus* (Schreber, 1775)—“gato-mirim”, little spotted cat, Oncilla	~ 2.5	Meat, tail, fat	Wounds, urinary incontinence in children, muscle injuries, throat problems, “estrepes” (suck a splinter out of skin), “to protect of bat attacks”, measles	VU	0.03
*Puma concolor* (Linnaeus, 1771)—“gato-açú”, “gato-maracajá-açú”, “onça bodeira”, “sussuarana”, Cougar, Puma	4	Fat	Throat problems, wounds	LC	0.02
*Puma yagouaroundi* (É. Geoffroy Saint-Hilaire, 1803)—“gato-vermelho”, “gato-azul”, Jaguarundi	~ 4	Fat	Wounds	LC	0.01
Mephitidae					
*Conepatus semistriatus* (Boddaert, 1785)—“tacaca”, “ticaca” Striped Hog-nosed Skunk	~ 3	Meat, tail, odoriferous anal gland, fat, bones	Arthritis, burcite, kidney stone, headache, heel spur (“esporão de galo”), throat inflammation, improve eyesight, spinal disorders, “to protect of bat attacks”, spinal disorders, osteoporosis, rheumatism, tuberculosis	LC	0.69
Myrmecophagidae					
*Tamandua tetradactyla* (Linnaeus, 1758)—“tamanduá”, “tamanduá-mirim”, Southern Tamandua	~ 7	Meat, leather, fat, bones, claw	Allergies, asthma, epilepsy, hemorrhoids, bleeding in women, inflammations, bronchitis, “to protect of snake bites”, rheumatism	LC	0.11
Procyonidae					
*Procyon cancrivorus* (G.[Baron] Cuvier, 1798)—“guaxinim”, Crab-eating Raccoon	~ 6	Tail	“To protect of snake bites”, “to protect of bat attacks”	LC	0.02

**Fig. 2:**
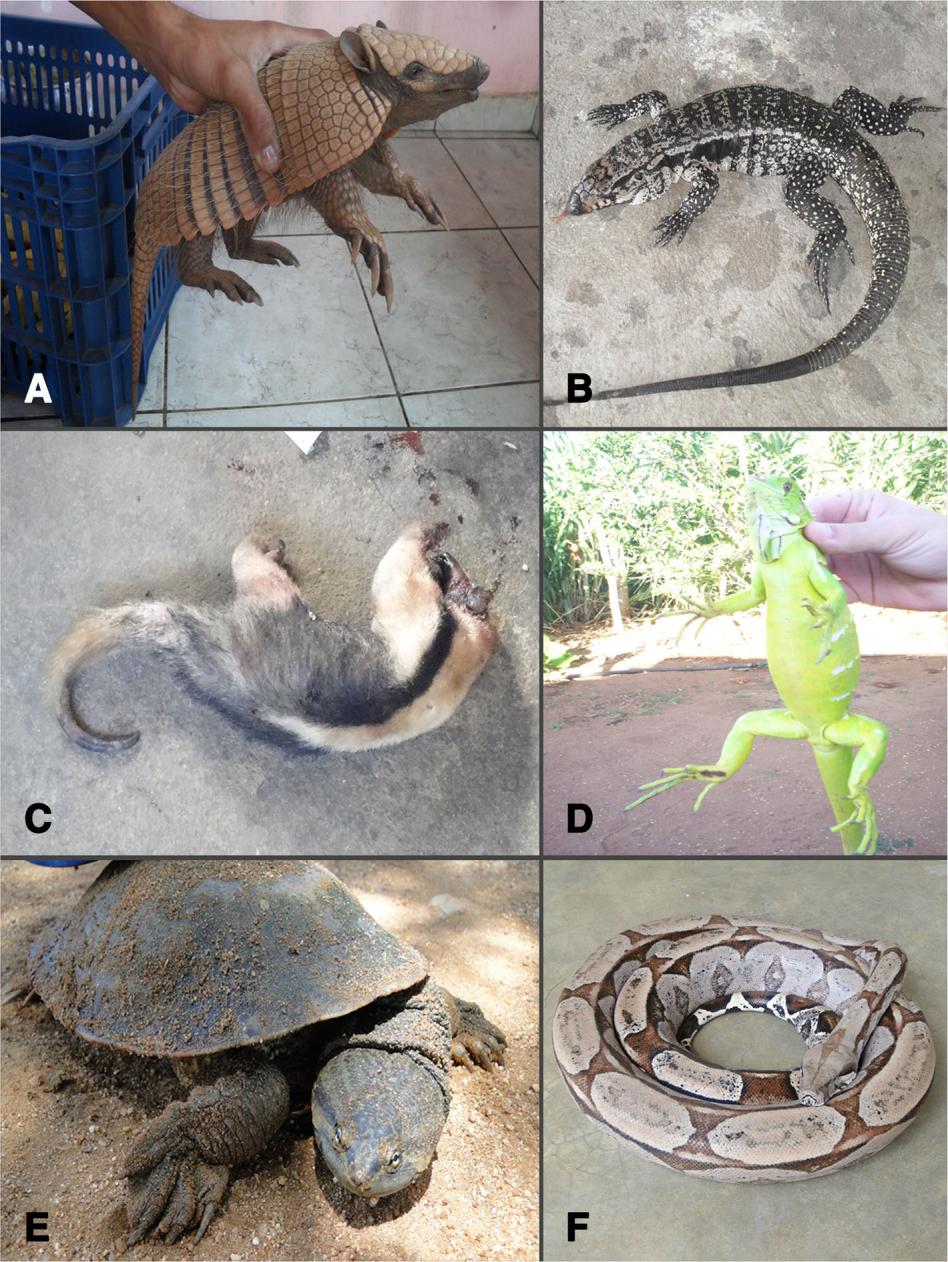
Examples of hunted/trapped species due to traditional medicine value in semiarid of NE Brazil. **a** *Euphractus sexcinctus*. **b** *Salvator merianae*. **c** *Tamandua tetradactyla* hunted for trade of zootherapeutic byproducts. **d** *Iguana iguana*. **e** *Phrynops tuberosus*. **f** *Boa constrictor*. Photos **a**, **b** Wallisson Sylas. **c**–**f** Wedson M. S. Souto

The average number of species hunted for medicinal purposes by each hunter was 5.4 ± 2.64, with aspects of socioeconomics influencing the number of target species. The MLR model revealed a significant, weak positive relationship (*R*^2^ = 0.073, ANOVA *F*_4,252_ = 4.97, *p* < 0.001) only between species richness and hunter age (Table 3). According to UV, the most important medicinal species were *Salvator merianae* (Duméril & Bibron, 1839) (UV = 1.09), *Conepatus semistriatus* (Boddaert, 1785) (UV = 0.69), *Mesoclemmys tuberculata* (Lüderwaldt, 1926) (UV = 0.53), *Crotalus durissus* Linnaeus, 1758 and *Phrynops tuberosus* (Peters, 1870) (UV = 0.52), *Cerdocyon thous* (Linnaeus, 1766) (UV = 0.48), *Boa constrictor* Linnaeus, 1758 (UV = 0.47), and *Iguana iguana* (UV = 0.44). Figure 2 provides examples of animals cited in the present study as sources of traditional remedies.

**Table 3:** Coefficients of the multiple linear regression model and explanatory power (*R*^2^) of hunters’ socioeconomic predicting variables and target-species richness

Predicting variables	*B*	SE *B*	*β*
Constant	4.03	0.58	
Hunters’ age	0.03	0.01	0.18*
Hunters’ zone of residence (urban, peri-urban)	0.45	0.33	0.08
Trade of zootherapeutic species (yes, no)	− 0.32	0.37	− 0.05
Education level (very low, medium/high)	0.61	0.43	0.11

Note: *R*^2^ = 0.073, *p* < 0.001. *p < 0.05

The hunting of species for traditional medicine was linked, in many ways, with exploitation for other purposes. For example, according to the interviewees, several local species are hunted mainly for food, and not just for their zootherapeutic value. This is particularly true for a number of bird species (e.g., *Sarkidiornis sylvicola*, *Tachybaptus dominicus*, *Penelope jacucaca*, all species of Columbidae, and many species of Tinamidae) and mammals (*Cuniculus paca*, Caviidae spp., Dasypodidae spp., *Thrichomys laurentius*, *Conepatus semistriatus* and *Tamandua tetradactyla*). Others, such as carnivores of the families Felidae and Canidae (e.g., *C. thous*), and the marsupial *Didelphis albiventris*, are usually hunted because of conflicts with humans (for instance, hunting or trapping of wild carnivores to avoid livestock predation), with the subsequently being used in traditional medicine.

Although there is a link between hunting for medicinal purposes and other uses of wildlife, including food, a variety of animal body parts harvested by hunters (e.g., bones, caruncles, feathers, gall bladders, leather, scent glands, tails, teeth, livers, and meat) were not commonly consumed as food. Our results, however, showed that meat was the most important zootherapeutic item obtained by hunters, with it being extracted for medicinal purposes from at least 19 species (Table 2). Animal fat, a highly sought-after medicinal resource harvested by hunters, is obtained from 17 species, including *Salvator merianae*, *Mesoclemmys tuberculate*, *Crotalus durissus*, *Boa constrictor*, and *Dasypus novemcintus*. Fat, feathers, leather, bones, teeth, and similar dried parts are usually stored in glass or plastic containers, thereby allowing their sale or use several days after extraction.

In general, species used for bushmeat were also versatile as traditional remedies. Twenty-six species (66.5%) were recorded to treat more than one disease or symptom. The most versatile species were *Iguana iguana* (used to treat 26 diseases or conditions), *Salvator merianae* (23), *Crotalus durissus* (20), *Kerodon rupestris* (19), and *Boa constrictor*, *Mesoclemmys tuberculata* and *Cerdocyon thous* (17 each). In addition, parts from several different animal species were prescribed for treating wounds, including *Rhinella jimi* (Stevaux, 2002), *Cerdocyon thous*, *Leopardus* spp., and *Puma* spp.

The preparation and administration of zootherapeutic resources often involves the toasting or powdering of hard animal parts or whole animals. The powder obtained is then used to prepare teas, is ingested with food, or is inhaled. Meat and viscera are used as both food and as medicinal resources, whereas fat and metabolic secretions are topically administered as ointments or ingested. The hunters described zootherapeutics for 92 diseases or conditions (Table 2), with wounds (with 16 species indicated for treatment), rheumatism (11), the removal of thorns (9), ear aches, and sore throats (8 each) being the principal conditions treated.

### Destinations of zootherapeutic animals and their conservation status

A total of 73.5% (*n* = 189) of the hunters reported selling wild animals or their byproducts for medicine purposes. Thirty-three (12.8%) of the hunters reported selling animal products to herbalists, while others (*n* = 156) stated that they sold directly to end consumers. The reasons reported for selling animal-derived remedies or whole specimens were diverse and included both subsistence (for example, earning money to buy food) and luxury (e.g., alcoholic beverages, cigarettes, smart phones, motorcycle parts) purchases and the defrayment of the cost of hunting expeditions.

The sale of animal parts or whole organisms to herbalists or final consumers usually occurred between 5:00 am and 7:00 am, after contacts were made via mobile phone for scheduling deliveries of the zootherapeutic products/specimens. The interviewees indicated that end consumers usually go to the hunters’ homes to acquire wild animals/parts; this was witnessed in the field by one researcher (WMSS). More recently (2015), informal conversations with hunters revealed that a number of them use social networks (e.g., Whatsapp©, Facebook©) to contact (or be found by) end consumers and herbalists. Wholesalers/middlemen were not mentioned by the hunters and were not detected by us (WMSS) in the market chain of zootherapeutics.

Hunting expeditions were reported to be regularly organized for capturing some species with medicinal value, such as hunting tegus (“*batida de tejus*”) during the rainy season, or *Nothura* spp. (“*codorniz*”). Hunters almost unanimously reported capturing zootherapeutic species during hunting expeditions intended for other purposes or targeting other animals. This finding demonstrates the importance of zootherapy as an alternative form of health care for local inhabitants, even in areas where health services are provided by the government.

Regarding the conservation status of the animals harvested, a total of 34 species were listed in the IUCN Red List version 2016-3 (Table 2), although only two were considered threatened to some degree. All mammals and virtually all birds listed here had hunting as a major threat. Most of the animals were not considered threatened because they possessed wide geographic distributions that increase the likelihood that some viable populations remain in areas sparsely populated by humans [38].

## Discussion

The broad range of hunters’ ages strengthens the view that cultural as well as socioeconomic factors serve as important motivations for hunting activities [16, 49, 50]. The weak relationship between hunter age and species richness exploited, together with a lack of significant explanatory power of other socioeconomic variables in relation to species richness of medicinal bushmeat, suggest that cultural factors and hunter and end-user preferences for certain wildlife products are drivers of the trade demand and the types of bushmeat targeted by each hunter. A similar situation was observed by Baia Júnior et al. [51] in the market chain of bushmeat products in the Brazilian Amazon. The consumption of wild species as remedies in several parts of Northeast Brazil, even when alternatives are available, reveals the cultural acceptance and economic importance of this practice, especially considering the growing context of urbanization in the region. As highlighted by Ferreira et al. [6], traditional medicine is connected to cultural and biological questions regarding animal trade, but socioeconomic aspects are also essential to the maintenance of this activity. In this context, whereas traditional wildlife uses persist with adaptations in parts of the Neotropics [52], the loss of local knowledge regarding therapeutic natural resources is also remarkable [53]. This is particularly significant considering that trade in medicinal animals represents one of the available options for livelihood for a portion of local populations [7, 53].

The high prevalence of hunters involved in wildlife trade for medicinal reasons is in line with previous studies performed in tropical regions that focused on end consumers or traders (e.g., herbalists, market sellers), which showed animal-based remedies to be widely consumed wildlife products in urban and peri-urban areas, and established trade networks that deal with the storing, transporting, and selling of products in places distant from where they were originally collected [19, 54, 55]. As with hunting bushmeat for food in other tropical areas [56, 57], the illegal trade of medicinal animals in the semiarid area of the Northeast Region of Brazil provides an opportunity for immediate cash income. The predominance of local hunters engaged in the market chain of zootherapeutic products is a clear picture of how the boundary between subsistence and commercial hunting is blurred [58, 59], with personal interests and socioeconomic realities operating together to stimulate wildlife harvesting [60].

The species richness of terrestrial vertebrates recorded in the present study was higher than that reported by previous field studies in urban or peri-urban areas of the Northeast Region of Brazil (see [19, 21]). Thus, it appears that these research, which only sampled end points of the traditional medicine market chain, missed records of wild animals targeted by the medicinal value or/and of the trade of traditional medicine resources.

There is evidence that the composition, accessibility, and availability of a local fauna directly influence the taxonomic groups and products used in traditional medicine in any given region [61]. Despite the species mentioned by hunters as being native of caatinga dry forest, the importance of mammals as sources of folk remedies has also been observed in communities, villages, and urban centers in humid or semiarid regions of Neotropical countries (see [1, 6, 19]), and peri-urban or urban communities in Africa [11, 55]. Wild mammals and reptiles are likely preferred targets of hunting for medicinal purposes due to the large quantities of parts that can be acquired from them and easily used and stored (including fatty tissue, flesh, and bone) [15]. In the present study, the average of body weight (AW) of bird species was 0.56 kg, while that of reptiles was 2.26 kg and that of mammals 4.18 kg (also Table 2). In addition, in the dry forest of the Caatinga, mammals and some reptiles represent the wild fauna most encountered throughout the year, since most birds typically migrate during the dry season (see Olmos et al. [62]) and only some small-sized birds (e.g., species of Columbidae) are relatively abundant and more easily found and hunted than mammals [63].

Certain taxa seem to be hunted because of cultural preferences. For example, some of the species hunted for purposes of traditional medicine in the semiarid area of the Northeast Region of Brazil are also popular sources of animal-based remedies in other parts of Brazil and South America. Species of the family Teiidae (*Salvator* spp. and *Tupinambis* spp.), for instance, are used as ingredients in popular medicines in Argentina, Bolivia, and other regions of Brazil [19, 64, 65]. Indeed, Bonifácio et al. [66] recently suggested that *Salvator merianae* is the major medicinal species for semiarid areas in Brazil. The results reported by these authors reinforce previous investigations about the use of wild fauna-based remedies in areas of Caatinga in Northeast Brazil (e.g., [4, 19, 67]), and highlight the popularity of this species as a medicinal resource in this region. Local communities have historical and transgenerational knowledge of the uses of some wild species. For example, species of the family Teiidae are well-represented among Brazilian pharmacopeia [15]. The use of *Salvator merianae*—as well as other species of Teiidae—in popular medicines demonstrates that this animal is locally and/or ecologically important within a cultural context and on a temporal scale [66]. The popular use of a species in a traditional pharmacopeia may ultimately represent a well-constructed system of exhaustive trial and error, which leads to the selection of animals considered useful for specific treatments. These species usually represent important subjects for pharmacological studies, as has been the case for *Salvator merianae* [68, 69].

Although a number of authors have noted that faunal constituents of traditional medicines are often byproducts of food animals that would otherwise be discarded (see [6, 19]), we did not observe this pattern in present research. The importance of wildmeat as a zootherapeutic remedy reinforces the need for further research at the level of the hunter about use and commercialization of wildlife in traditional medicine. Intensive use of meat was clearly detected in this study because hunters are the key harvesters of this resource (see [16, 70, 71]). Other animal parts used in traditional medicines in Brazil, in particular fat of the lizards tegu and *Iguana iguana* and the snakes *Boa constrictor* and *Crotalus durissus*, can be easily found as therapeutics in public markets or delivered to order for use in traditional medicine [21, 72]. Studies have shown that these fats can reduce inflammatory processes [68]; however, the therapeutic role of meat has been neglected and is in need of further pharmacological investigation.

As has been reported in Brazil, and other countries where wild animal trade is fully or partially illegal [15, 54], most intermediaries of medicinal animal trade do not directly exhibit animals or animal parts for public viewing, and the ones occasionally seen for sale at markets likely represent only a tiny portion of that which is traded in a more clandestine manner. Our results also support the findings of studies [19, 21, 73] performed with final consumers and market vendors (herbalists), which found that less perishable animal parts are commonly found at these levels of the market chain because they can be processed into products more easily hidden or camouflaged in markets (e.g., bone powder, small leather pieces, animal fat in small bottles).

The notable lack of middlemen between hunters and end consumers or market vendors distinguishes local zootherapeutic trade dynamics in the semiarid area of the Northeast Region of Brazil from other bushmeat trade operations for food or medicinal purposes (e.g., [54, 74]). Based on data from field research and the literature (W.M.S.S., unpublished data [75]), we believe that middlemen are being eliminated from the local market chain by synergistic factors, which include (1) increased availability of personal transportation (especially motorcycles), which facilitates direct exchanges between hunters and their customers; (2) increased availability of mobile phones that facilitate direct contact between consumers/herbalists and hunters, even when separated by considerable distances; and (3) low commercial values of zootherapeutics compared to other wildlife products in Brazil (e.g., bushmeat, wild pets). Therefore, the illegal trade of medicinal animal parts represents a true “fast ghost market”.

The availability of zootherapeutic species is assured for hunters and the local demand despite the climatic seasonality typical of the Caatinga domain. Coupled with the use of dry parts of wild animals in traditional medicine, Ferreira et al. [6] emphasized the existence of versatile species in favoring the continued demand for zootherapeutics by satisfying consumer demands seamlessly throughout the year, with the availability and accessibility of wild animal medicines in any given season being generally assured because a number of species can be used to treat the same disease.

Although there are only a few target species that are currently threatened, this should not be taken to mean that traditional medicine has a minimal impact on populations of zootherapeutic fauna. As highlighted by others [6, 19, 76], hunting to harvest medicinal species creates additional continuous pressure on wild populations. For the White-browed Guan, *Penelope jacucaca*—a vulnerable cracid included in a Brazilian plan for bird conservation [77]—or for a few species of carnivores (e.g., wild Felidae and Canidae spp.), the removal of any individuals from their natural habitats creates even greater conservation concern [78, 79].

The adoption of new conservation strategies that reconcile local demand with low levels of hunting for medicinal, or other, uses is needed [80]. There is growing recognition that approaches that employ radical prohibitions only, such as the current total hunting ban in Brazil, are insufficient for curbing hunting and poaching [80, 81]. Efficient conservation policies will require adopting both biological and socioeconomic perspectives in their implementation strategies [82]. Wildlife farming, for example, offers a potential strategy for meeting local demands while minimizing the number of wild zootherapeutic animals taken in the region of the Caatinga. In order to successfully breed species used in traditional medicinal practices in captivity, it will be necessary to provide instruction to breeders and local residents of the necessity of honesty in their dealings (together with efficient local law enforcement agencies) to avoid breeding farms from acting simply as covers for introducing illegally captured wild animals into the market [83].

It is important to emphasize that wildlife farming has been established as a profitable activity in some countries [84, 85]. The monetary return from wildlife production from captive breeding systems is indeed very high for some species. For instance, investment in high-value game breeding in 2008 would have resulted 187% return on investment by the end of 2012 in South Africa [85]. Nonetheless, wildlife farming cannot be regarded as a sole strategy for wildlife conservation because of ecological, social, and economic consequences. In general, breeding production is a market-oriented activity and, thus, as a rule, only high-production species (e.g., r-strategists) or species of high profitability are incorporated into ranching strategies [85, 86]. As a consequence, non-economically important wild animals may eventually be marginalized in the process of implementing and expanding wildlife farming, resulting in intensified human-wildlife conflicts (e.g., human vs. wild carnivore conflicts). In this way, game ranching may become less compatible with broad-spectrum species conservation [87].

Based on comparative data for species targeted for breeding, Tensen [88] recognized specific criteria by which wildlife farming can benefit species conservation: (1) production cost must be less than that for wildlife black markets, (2) restocking of farms with wild specimens must not take place, (3) laundering of wildlife into legal markets must be non-existent, (4) a substantial portion of local demand for wildlife products is met by farming wild products, and (5) consumers must show a strict preferences for captive-bred specimens only. Criteria (4) and (5) in particular are key problematic points from cultural and economic perspectives. As stated by van Vliet et al. [86], the ever-increasing human population and the high demand for wild products justifies the exploration of opportunities for the production of meat and other byproducts of native species. Nonetheless, the repertoire of vertebrates known to have effective potential for captive production in South America is small, including *Salvator merianae* [89–91]. The costs associated with investments into the implementation, maintenance, and marketing of wildlife farming systems are very high for small- or medium-scale producers [86]. This reality competes with what is recognized as one of the most biodiverse faunas in the world, which is by far more inexpensive and accessible for commercial and subsistence hunters [86]. The viability of a diversity of wildlife farming programs in South America is broadly dependent on governmental and/or non-governmental agencies being persistently involved in subsidizing establishment of suitable places for ranches, provisioning technical assistance, and introducing captive breeding centers to supply founder stock [89].

Although we do not have quantitative data about frequency of harvesting and consumption of species for medicinal purposes, based on the information of interviewees that hunting of the most popular species used in the local traditional medicine system is common in the surveyed areas, as well as data of other localities of North and Northeast Brazil (see [6, 21, 61]) which suggest a wide-ranging trade of wild animals for traditional medicine, it is unlikely that there is a lower level of hunting and trapping for medicinal purposes, especially for wild animals primarily harvested for meat consumption. In addition, major hunting techniques encompass dogs, firearms, and traps as described by Barboza et al. [70], Bezerra et al. [92], and Souza et al. [16]. This varied repertoire of hunting methods has been reported as efficient in the Neotropical region and associated with areas with intense faunistic exploitation [70, 93, 94].

We believe that the use of bushmeat and other wild animal products as traditional medicine is influenced in different ways by social networks. It is impossible to unlink the activities of a local community from urban reality when implementing policies are aimed at resolving problems associated with wildlife exploitation. Local preferences contribute to the construction of strategies that may enable local demand to obtain easier access to wildlife resources. There are several ways by which social networks are associated with wildlife consumption, the most popular of which is the use of wildlife products as gifts for relatives, friends, and locally important people in both rural and urban contexts (see [95, 96]). Given that zootherapeutic products are popular in both large and small cities in Northeast Brazil [6, 19] and that hunters and their relatives are also consumers of zootherapeutic remedies (this study and Policarpo et al. [7]), it is likely that such products are also interchangeable, exchanged, or even given as an item to family members, trusted people, or friends.

Removing the black trade of zootherapeutic products from market places requires some degree of trust between local clients and hunters. Hunters are unlikely to offer animals for sale to people that they do not trust or who have not been recommended by trusted people. As seen by others, especially where trade in wildlife and it byproducts is illegal and law enforcement is not negligible, the relationship between suppliers of wild animal-based remedies and their customers is defined by mutual trust and rapport [97, 98]. Thus, we also recognize that there is a well-established social relationship between suppliers of herbalists and local hunters in the surveyed areas. Social/trust networks from three perspectives (hunters—herbalists; hunters—end customers; and hunters as zootherapeutics users—other friends/relatives) enable, in a multitude of ways, the illegal distribution of wild vertebrates. This, in turn, implies that there could be considerable hidden and specialized local and regional trade in wildlife [98].

Better understanding of consumer demand and drivers of the use of medicinal animals, as already done in studies on bushmeat for food purposes [70, 86, 96], are needed for more efficient strategies for wildlife conservation. Simple top-down conservation models have proved inefficient in tropical contexts when considered as unique strategies for conservation [99]. In Brazil and other Latin American countries, despite popular knowledge about illegality of hunting and trapping, exploitation of wild species is widespread for both urban and rural contexts. As there are social groups with dependence on the use and trade of medicinal animals for subsistence purposes, as well as these products are culturally popular, conservation policies should also consider promoting the use and trade of culturally accepted and proven effective traditional medicines. Where it is indispensable for subsistence of local communities, participatory hunting management, depending on kind of target species, can still be considered as one of the multiple and concomitant strategies for the use and conservation of the wild fauna (see [56]).

## Conclusions

This present hunter-level survey illustrated that there is still much to be learned about the dynamics of illegal wildlife trade. Zootherapy is usually neglected as a driver for hunting in the neotropical region. However, our study shows just the opposite. The hunting of wildlife for medicinal purposes is a culturally motivated activity which supplies personal demands of hunters, as well as urban users.

Additionally, hunters provided details of an undetectable scenario in previous studies on zootherapy in Brazil. In addition to a rich diversity of wild vertebrates exploited for medicinal purposes, the hunter-level data show that the hunting for medicinal purposes in the semiarid region of Brazil is mostly linked to hunting for bushmeat. Our study demystifies the idea of an illegal wildlife trade traditionally based on physical markets (e.g., street/open fairs), since the incorporation of technological resources enabled hunters to reach out to end consumers by eliminating middlemen and flourishing an even darker market. Consequently, there is a need for a reassessment of the wildlife hunting and trade scenario for the elaboration of more efficient and participative conservation strategies by Brazilian authorities.

As some of the zootherapeutic species used in Brazilian traditional medicine appear to have true pharmacological potential [68], and local residents will not desist from purchasing natural resources for their health care, the development of a legal regulatory mechanism for the acquisition and use of animals in folk medicine is urgently needed, together with studies to evaluate the pharmacological validity of zootherapeutic medicines and the risks involved in their use. Conservation research focusing on game species and identifying culturally acceptable alternatives based on plants or domestic animals are also necessary.
